# Clinical outcomes and kinetics of propanil following acute self-poisoning: a prospective case series

**DOI:** 10.1186/1472-6904-9-3

**Published:** 2009-02-16

**Authors:** Darren M Roberts, Renate Heilmair, Nick A Buckley, Andrew H Dawson, Mohamed Fahim, Michael Eddleston, Peter Eyer

**Affiliations:** 1 South Asian Clinical Toxicology Research Collaboration, University of Peradeniya, Peradeniya, Sri Lanka; 2 Medical School, Australian National University, Canberra, Australia; 3 Department of Clinical Medicine, Faculty of Medicine, University of Colombo, Colombo, Sri Lanka; 4 Walther-Straub Institute of Pharmacology and Toxicology, Ludwig-Maximilians University, Munich, Germany; 5 Faculty of Medicine, University of Peradeniya, Peradeniya, Sri Lanka; 6 Scottish Poisons Information Bureau, New Royal Infirmary, Edinburgh, UK

## Abstract

**Background:**

Propanil is an important cause of death from acute pesticide poisoning, of which methaemoglobinaemia is an important manifestation. However, there is limited information about the clinical toxicity and kinetics. The objective of this study is to describe the clinical outcomes and kinetics of propanil following acute intentional self-poisoning.

**Methods:**

431 patients with a history of propanil poisoning were admitted from 2002 until 2007 in a large, multi-centre prospective cohort study in rural hospitals in Sri Lanka. 40 of these patients ingested propanil with at least one other poison and were not considered further. The remaining 391 patients were classified using a simple grading system on the basis of clinical outcomes; methaemoglobinaemia could not be quantified due to limited resources. Blood samples were obtained on admission and a subset of patients provided multiple samples for kinetic analysis of propanil and the metabolite 3,4-dichloroaniline (DCA).

**Results:**

There were 42 deaths (median time to death 1.5 days) giving a case fatality of 10.7%. Death occurred despite treatment in the context of cyanosis, sedation, hypotension and severe lactic acidosis consistent with methaemoglobinaemia. Treatment consisted primarily of methylene blue (1 mg/kg for one or two doses), exchange transfusion and supportive care when methaemoglobinaemia was diagnosed clinically. Admission plasma concentrations of propanil and DCA reflected the clinical outcome. The elimination half-life of propanil was 3.2 hours (95% confidence interval 2.6 to 4.1 hours) and the concentration of DCA was generally higher, more persistent and more variable than propanil.

**Conclusion:**

Propanil is the most lethal herbicide in Sri Lanka after paraquat. Methylene blue was largely prescribed in low doses and administered as intermittent boluses which are expected to be suboptimal given the kinetics of methylene blue, propanil and the DCA metabolite. But in the absence of controlled studies the efficacy of these and other treatments is poorly defined. More research is required into the optimal management of acute propanil poisoning.

## Background

Propanil (3,4-dichloropropionanilide) is a selective acylanilide herbicide used widely in rice cultivation in many parts of the world. It may be the most extensively used herbicide for rice production worldwide [1] and is ranked within the top 20 pesticides used for agriculture in the United States.[2] Unfortunately, acute self-poisoning leading to severe poisoning and death has been reported with propanil, particularly in Asia where subsistence farming is more common. [3-10] Death is largely attributed to severe methaemoglobinaemia that is prolonged and treatment-resistant. It is not known whether these published cases are representative of the usual outcome from acute propanil poisoning or why current medical care is of limited efficacy.

**Figure 1:**
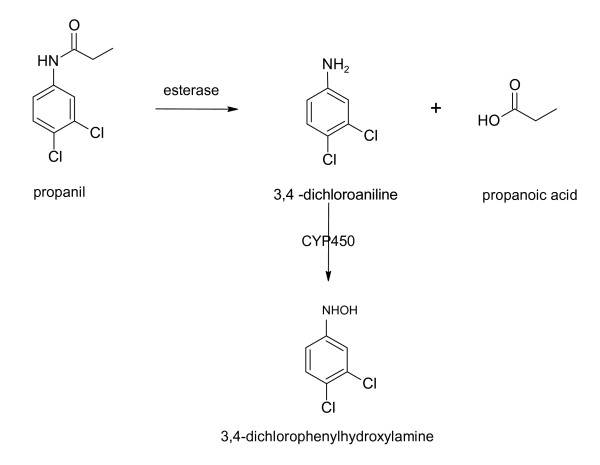
**Metabolism of propanil *in vivo*, including the clinical toxicity associated with each metabolite**. [11-15]*. * Propanil (CAS No. 709-98-8) is also classified as a chloroaniline herbicide and known as DCPA, propanide and *N*-(3,4-dichlorophenyl)propanamide. It is stable in solution at pH 3 – 9 but may be subject to hydrolysis to 3,4-dichloroaniline and propionic acid outside this range, although this has been debated. If hydrolysis occurs to a significant extent in the acid medium of the gastrointestinal tract there may be a diminished importance for esterases for metabolism of propanil. The specific esterases that hydrolyse propanil to DCA have not been identified but are known to be inhibited by paraoxon and sodium fluoride. A minor metabolite of CYP450 oxidation of DCA is 6-hydroxy-dichloroaniline which is less toxic.[13].

Propanil is hydrolysed *in vivo *to 3,4-dichloroaniline (DCA) which is in turn oxidised to 3,4-dichlorophenylhydroxylamine which is a potent inducer of methaemoglobin, figure 1. [11-15] These reactions are similar to those of dapsone which are well characterised: the severity of methaemoglobinaemia relates to the amount of dapsone's hydroxylamine metabolite, which varies with dose and cytochrome P450 activity.[16,17] We therefore expect that there will be a proportional relationship between the concentration of propanil and its metabolites and clinical toxicity, although this has not yet been confirmed in humans.

The objective of this study is to describe the clinical outcomes of acute intentional self-poisoning from propanil in a large, multi-centre prospective cohort study in general hospitals in Sri Lanka. The relationship between the admission concentration of propanil and 3,4-dichloroaniline, and some details on their kinetics will also be assessed, in particular their relationship to clinical toxicity.

## Methods

### Clinical

The South Asian Clinical Toxicology Research Collaboration is conducting clinical studies on acute poisoning in Sri Lankan rural hospitals where the incidence of intentional self-poisoning is high.[18] Patients were included in this analysis if they presented to a study hospital with a history of acute propanil poisoning. Most patients were recruited from Anuradhapura Teaching Hospital (187 consecutive patients between 5^th ^April 2002 and 13^th ^April 2007) and Polonnaruwa General Hospital (242 consecutive patients presenting between 7^th ^June 2002 and 7^th ^April 2007). These patients presented to each hospital directly or via transfer from a district hospital where they had been medically reviewed. Resources are limited in these rural hospitals, however, they do provide 24-hour medical and nursing care to patients in dedicated general medical wards.

Patients were identified by on-site study doctors on presentation. Following the initial clinical assessment all patients provided an admission blood sample which was used to confirm the exposure reported on history. Some patients provided additional blood samples because they were recruited into one of two studies:

1 RCT1: ISRCTN02920054 is a randomised controlled trial (RCT) evaluating the efficacy of oral superactivated charcoal in acute poisoning. Patients were allocated to a regimen of superactivated charcoal (single dose activated charcoal, multiple doses of activated charcoal, or no activated charcoal) using a stratified randomisation protocol based on time since poisoning and clinical toxicity on admission. Propanil was included with other miscellaneous pesticides during randomisation. This study was completed in 2005 and did not demonstrate a clinical benefit from charcoal.[19]

2 Pharmacokinetic (PK) study: This study followed RCT1. Patients with acute pesticide poisoning presenting to study hospitals consented to provide multiple blood samples during admission, as detailed below. Patients were administered activated charcoal at the discretion of the treating physician.

Ethics approval for the above studies was obtained from the Universities of Colombo (Sri Lanka), Oxford (United Kingdom), Peradeniya (Sri Lanka), Australian National University (Australia) and/or Sri Lankan Medical Association (Sri Lanka). Written informed consent was obtained for the two studies with multiple blood sampling.

The history of exposure (including co-ingestants) and clinical details were obtained on presentation for each patient. Patients were regularly reviewed and prospectively determined clinical details were recorded by on-site study doctors until discharge or death. Severity of poisoning was graded on the basis of predetermined clinical criteria using a simple system (table 1). Co-oximetry for quantification of methaemoglobinaemia was not available for the duration of the study given resource limitations in these rural hospitals. Blood gas analysis was available for a short period of time in one of the study hospitals and these measurements were used to support clinical management.

**Table 1:** Clinical grading of severity of acute propanil poisoning

Minor	Asymptomatic or mild symptoms (usually gastrointestinal or sedation) with stable vital signs and no other organ involvement
Moderate to severe	Poisoning requiring intervention (eg. clinical evidence of cyanosis, hypotension (MABP < 70 mmHg), hypoventilation requiring intubation, cardiac arrest or evidence of ischaemia, sedation or coma (GCS < 10), seizures, oliguria)

Death	

To obtain additional clinical information on the patients who died, the medical notes were retrospectively reviewed using a pre-designed data collection sheet by a clinical trial coordinator at each centre.

The initial treatment of all patients with acute pesticide poisoning consisted of resuscitation and gastrointestinal decontamination. Patients were monitored clinically (including pulse oximetry) and received supportive care including supplemental oxygen, intravenous fluids and ventilatory and haemodynamic (dopamine) support as required. In the absence of co-oximetry analysis, significant methaemoglobinaemia was diagnosed when there was cyanosis that did not correct with high-flow oxygen and ventilatory support in patients with a history of propanil poisoning. Brown-red coloured blood was usually noted if blood tests were obtained.

Treatment of methaemoglobinaemia was similar to local guidelines.[20] This consisted initially of methylene blue (methylthionium chloride) 1 mg/kg intravenous bolus for 1–2 doses. This was followed by exchange transfusion (infusion of one unit of cross-matched erythrocytes together with removal of one unit from the patient) for persistent clinical toxicity. In some patients laboratory grade methylene blue was administered orally because the intravenous formulation was unavailable; oral ascorbic acid (1 g two or three times daily) was also administered to some patients. These oral treatments were commenced irrespective of whether they were administered activated charcoal. Antibiotics (usually penicillin and metronidazole) were given when aspiration pneumonitis was suspected clinically.

All interventions were determined by the treating medical team irrespective of the patient's involvement in the studies. For the duration of these studies there were no major changes in clinical management of patients with acute propanil poisoning except that intravenous methylene blue was intermittently unavailable. Given the heterogeneity of these patients (in particular the variable exposures) and because this is an observational study, only conservative interpretations of the efficacy of these treatments are possible.

In addition to the admission blood sample, serial samples were provided by a sub-group of patients enrolled in RCT1 and all patients enrolled in PK study. Blood samples were obtained on admission to hospital, then at 1, 4, 12, and 24 hours after initial clinical assessment, then once daily until discharge or death, as allowed by clinical factors. Samples were collected into an EDTA tube which was promptly centrifuged and the plasma was removed and frozen at -23°C until analysis of the concentration of propanil and 3,4-dichloroaniline.

### Laboratory

Laboratory analyses were conducted at the Walther-Straub Institute of Pharmacology and Toxicology, Ludwig-Maximilians University, Germany. Propanil and DCA (Pestanal^® ^quality) were purchased from Riedel de Haen, Seelze, Germany. Stock solutions (10 mM) were prepared in 40% CH_3_CN/60% 10 mM ammonium acetate, pH 4.5 (v/v) and mixed with blank heparinized plasma to give a final concentration of 40 μM propanil and 4 μM DCA.

Plasma samples (100 μL) were treated with CH_3_CN (150 μL) for 15 min to minimize protein binding, followed by centrifugation at 10 000 rpm for 15 min. After addition of 3 M trichloroacetic acid (50 μL) the mixture was vortexed and centrifuged for another 15 min. 200 μl of the supernatant was adjusted to pH 5 with 16 μL of a mixture of conc. ammonium hydroxide and glacial acetic acid (2:1, v/v). 20 μL of this mixture was subjected to HPLC on LiChroSpher^® ^100 RP 15 (5 μm; 125 mm × 4 mm I.D.) with a guard column LiChroCart^® ^4-4 (Merck, Darmstadt, Germany). Isocratic elution was performed with 40% CH_3_CN/60% 10 mM ammonium acetate, pH 4.5 (v/v) at a flow rate of 1 mL/min and detection at 250 nm. Quantification was achieved by comparison of the peak areas of authentic plasma standards. The retention times of DCA and propanil were 8.0 min and 9.4 min, respectively; the limit of quantification (LOQ) was about 1 μM each.

### Kinetics and the dose-response relationship of propanil and DCA

The kinetics of propanil and DCA were determined in serial blood samples. The method of Beal was used to incorporate concentrations below the LOQ (between 0 and 1 μM).[21] Here, the first concentration value below the LOQ was fixed to 0.5 μM (0.5 × LOQ) and subsequent samples were excluded from further analysis. Patients in whom the concentration was not greater than the LOQ in at least two samples were excluded from the kinetic analysis. The medical notes of the patients included in this sub-study were reviewed (where available) to determine whether exchange transfusion was administered since this treatment has the potential to decrease the plasma concentration unrelated to endogenous clearance.[22]

Changes in the concentration of propanil and DCA during admission were described for individual patients who provided a sample on four or more occasions. An averaged description of this relationship for the population was then determined by comparing the ratio of propanil:DCA concentrations for all samples and plotted relative to the time post-ingestion. Most of these initial samples were obtained on admission or soon after, prior to treatments such as exchange transfusion.

To determine the apparent elimination half-life for the cohort as a whole, data obtained from all patients who provided serial blood samples (more than two) were collated. Data from each patient were plotted on a semi-logarithmic graph to determine if changes in concentration were first order and the extent of variation within this cohort. The best-fit apparent elimination half-life and 95% confidence interval was then determined by non-linear regression. This was performed by global fitting of the rate constant in a monoexponential decay model (C_t _= C_i_*exp(-k.t); where C_i _is the initial concentration and C_t _is the concentration after time t when elimination occurs with a rate constant of k). These analyses were performed using GraphPad Prism version 4.03 for Windows, GraphPad Software, San Diego CA USA. For propanil, only data following an apparent Cmax were included in this regression.

The dose-response relationship was demonstrated graphically by plotting the available concentration-time points relative to the clinical outcome for the patient (table 1) for both propanil and DCA.

### Statistical analyses

Statistical analysis compared baseline clinical and demographic characteristics for patients in three groups: mild poisoning, moderate-severe poisoning, and fatal poisoning, as defined in table 1. The chi-square test was used for categorical variables and continuous nonparametric variables were compared using the Kruskal-Wallis test with Dunn's multiple comparison test if P < 0.05. Receiver-operator characteristic (ROC) curves were constructed to demonstrate the sensitivity, specificity and likelihood ratios of threshold values from clinical and demographic variables for predicting death compared to survival. 2 × 2 tables were constructed to calculate sensitivity and specificity at the best threshold (as determined by Youden's index). Sensitivity is defined as the proportion of people who died that were predicted to die, and specificity as the proportion of people who survived that were predicted to survive. Positive and negative likelihood ratios and predictive values were also calculated. All analyses were conducted using GraphPad Prism and P < 0.05 was considered statistically significant.

## Results

There were 431 presentations with a history of acute propanil poisoning over five years (5^th ^April 2002 to 13^th ^April 2007). Of these patients, 40 had ingested at least one other poison (30 of these were poisoned by a combination product containing propanil and another agent, usually clomazone, fentrazamide, pendimethalin or thiobencarb) and 3 of these died. Patients with co-ingestions were not considered further.

A total of 356 blood samples were available for inclusion in this study, including serial samples in 26 patients, allowing kinetic analyses to be conducted.

### Clinical outcomes

**Figure 2:**
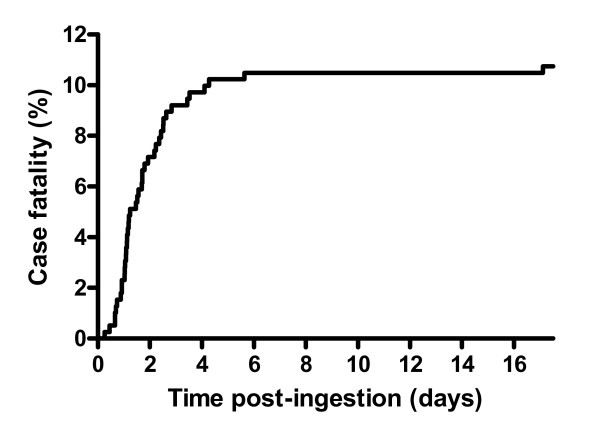
**Survival curve in patients with acute propanil poisoning**.

**Figure 3:**
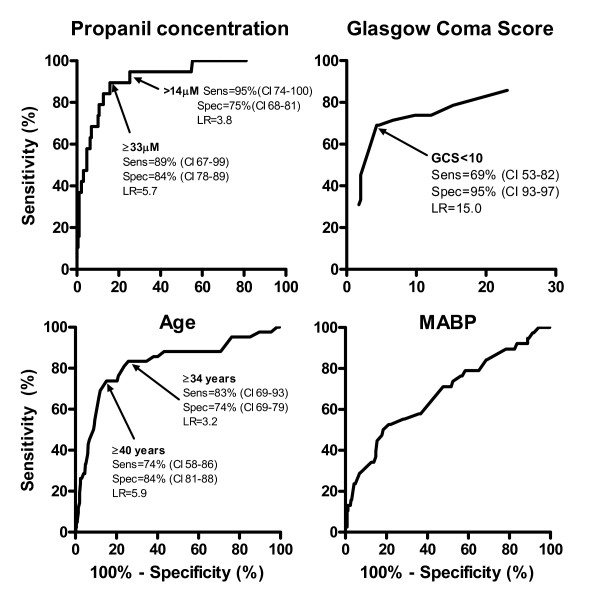
**Receiver-operator characteristic curves for predicting death from clinical and demographic information at the time of admission. (Sens = sensitivity, spec = specificity, CI = 95% confidence interval, LR = likelihood ratio)**.

There were 42 deaths (median time to death 1.5 days; IQR 1.0–2.4 days) giving a case fatality of 10.7% (42/391; 95% confidence interval 7.9–14.1%). Death occurred at staggered times post-ingestion which may relate to the amount ingested (where larger ingestions died earlier, although accurate dose information was not available) with the majority occurring within 3 days, figure 2. Patients who died were older, had a depressed GCS on admission, and a higher concentration of propanil in the admission blood sample (table 2). This was confirmed by the ROC curves (figure 3) with areas under the ROC curve of propanil concentration = 0.92, GCS = 0.88, age = 0.82. Values predicting death were determined and their corresponding sensitivity, specificity and likelihood ratios were calculated (figure 3). The initial mean arterial blood pressure and time to presentation did not appear to relate to the clinical outcome (table 2 and figure 3); for blood pressure, the area under the ROC curve = 0.68.

**Table 2:** Clinical outcomes, demographics and clinical features on admission

Severity of poisoning	Minor	Moderate to severe	Death	Statistical significance§
N/Total (%)	225/391 (57)	124/391 (32)	42/391 (11)	
Gender (M:F)	139:86	64:60	34:8	P = 0.003
Age (years)	24 (20–34)*^$^	22 (18–33)*^$^	45 (37–54)^$#^	^$#^P < 0.001; *P > 0.05
TTP (hours)	4.6 (2.7–-7.1)	5.3 (3.4–8.8)	4.5 (3.4–5.8)	P = 0.1431
GCS	15 (15-15)*^$^	15 (12–15)*^$^	7 (3–13)^$#^	^$# ^*P < 0.0001
MABP (mmHg)	89 (82–97)^$#^	83 (76–90)*^$^	78 (72–86)*^#^	^$#^P < 0.001; *P > 0.05
TTD (days)	2.2 (1.7–3.1)*	3.3 (2.6–5.0)*	1.5 (1.1–2.4)	*P < 0.001
Admission propanil concentration (μM)	1.3 (0.0–7.7)^$^* (n = 125)	8.9 (2.8–35.2)^#^* (n = 67)	72.0(45.8–128.3)^#$^ (n = 19)	*^$#^P < 0.001

Median values and inter-quartile ranges (IQR) presented

§Superscript symbols (*, ^$^, ^#^) are used to link two values with their respective P-value.

TTP = time to present TTD = time to discharge or death

Glasgow Coma Score (GCS) and the mean arterial blood pressure (MABP) are at the time of presentation to hospital

Nausea, vomiting, diarrhoea, tachycardia, dizziness and confusion occurred but were not a prominent feature in patients who did not develop severe poisoning. Some patients initially demonstrated central nervous system depression in the absence of documented hypoxia or cyanosis. Respiratory distress in the context of hypoxia on pulse oximetry with focal respiratory crepitations soon after presentation was considered to be aspiration pneumonitis; chest x-rays were not available to confirm this. Four of the patients who died were asymptomatic on admission but developed severe poisoning within 12 hours of ingestion and died between 1.2 and 2.4 days post-ingestion.

Patients with severe poisoning demonstrated depression of the central nervous system, hypotension and were clinically cyanosed. Death occurred in these patients following progressive hypotension in the context of cyanosis with low oxygen saturation on pulse oximetry (SpO_2 _~82–85%), despite active management. Blood samples were noted to be brown-red in colour. Blood gas analyses demonstrated severe lactic acidosis despite normal arterial oxygenation and hypocarbia consistently, suggesting tissue hypoxia due to severe methaemoglobinaemia with compensatory hyperventilation. Therefore, methaemoglobinaemia appeared to be an important manifestation of acute propanil poisoning.

### Management

Patients were administered variable regimens of methylene blue, ascorbic acid and exchange transfusion. Occasionally, methylene blue was given in the absence of clinical cyanosis at the time of admission, which may reflect the clinicians' anxiety regarding the potential for severe poisoning from such an exposure. There is a perception amongst some clinicians in Sri Lanka that methylene blue is of low efficacy for the treatment of acute propanil poisoning.[10] This is reflected in the records of patients who died, where only rarely were more than two doses of methylene blue given to patients with severe poisoning. Instead, exchange transfusion was observed to be applied more vigorously. The typical regimen was exchange of 1 unit every 1–2 hours until recovery or death, although most patients only received two or three exchanges. In some patients there was deterioration in blood pressure and respiratory failure following transfusion which might relate to an adverse reaction of the transfusion but it might also reflect the natural history of severe poisoning following a massive ingestion.

### Kinetics and the dose-response relationship of propanil and DCA

**Figure 4:**
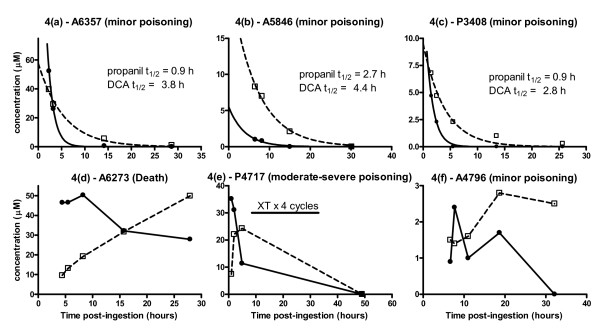
**Concentration-time profile of propanil and DCA in patients during admission**. DCA is indicated by open squares with dashed line and propanil is indicated by closed circles and solid line. Note that the scales differ; XT = exchange transfusion.

The concentration-time profiles of propanil and DCA for six patients with the most samples are shown in figure 4. First order elimination is noted in figures 4(a) to 4(c) and the half-life of DCA is longer than propanil. The profile of the lower three patients is less predictable. Allowing for a lag-time, changes in the concentration of DCA often parallel those of propanil, as observed in A4796, P3408 and P4717. Inter-individual differences in the apparent time-lag between these changes may reflect varying activities of the esterase required for metabolic activation (figure 1). The highest concentration of DCA generally exceeded that of propanil and remained elevated for a longer period. Most patients demonstrated a nonlinear concentration-time curve for propanil and DCA on the semilogarithmic graph, which may be consistent with ongoing absorption; exceptions are shown in figure 4. Data on exchange transfusion were only available in three patients in whom no substantial change in elimination was noted.

**Figure 5:**
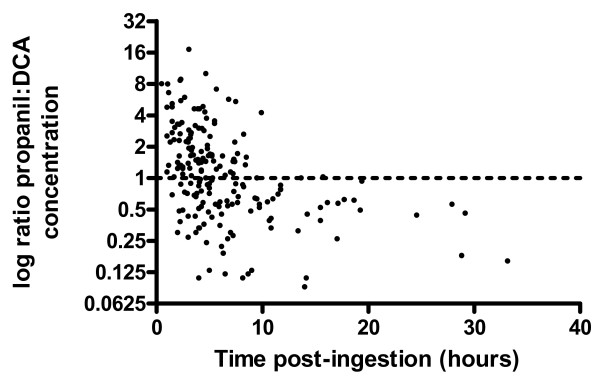
**The ratio of propanil:DCA concentration relative to time**.

Both propanil and DCA were quantified in 300 samples allowing for temporal changes in the ratio of propanil:DCA to be determined as shown in figure 5. It is observed that prior to 10 hours post-ingestion the ratio varies widely, which is probably due to ongoing propanil absorption and bioconversion to DCA. After 10 hours, however, the ratio is consistently less than 1.0 meaning that the concentration of DCA is higher than propanil. This could be due to propanil having higher clearance or a larger volume of distribution (Vd) than DCA. However, the observed half-life of DCA may also be strongly influenced by the rate of formation from propanil (a type of 'flip-flop' kinetics[22]).

**Figure 6:**
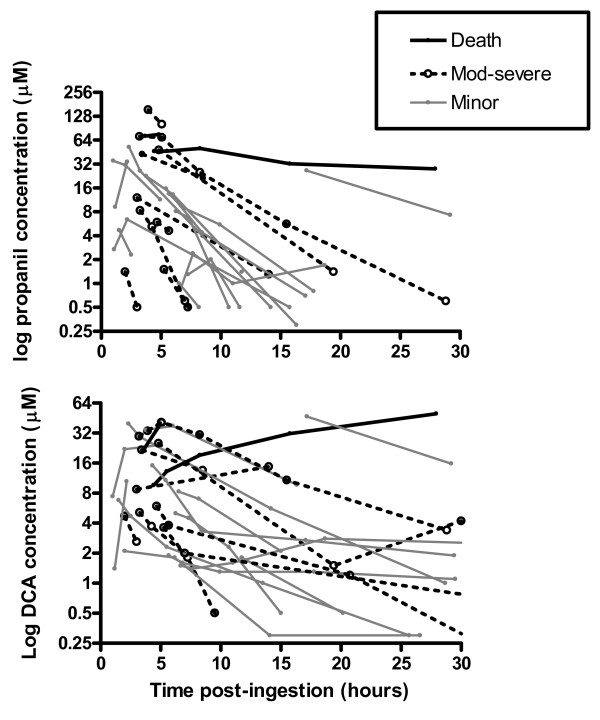
**Comparison of individual concentration-time profiles of propanil and DCA, including the clinical outcome**. The median best-fit apparent elimination half life of propanil = 3.2 hours (95% confidence interval 2.6 to 4.1 hours). Two of these patients were known to receive exchange transfusion and their half-life did not appear to differ from the others.

**Figure 7:**
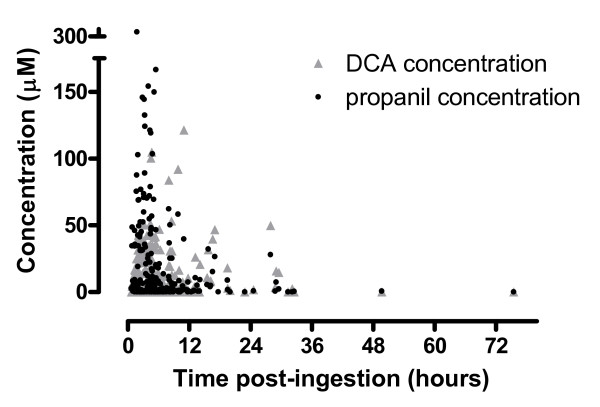
**All concentration-time points in patients with acute propanil poisoning**.

The concentration-time profiles of propanil and DCA for 26 individuals in whom serial samples were obtained are shown in figure 6 relative to their clinical outcome. Many patients demonstrate ongoing absorption of propanil until around 10 hours post-ingestion which was followed by an elimination phase. In survivors, by 36 hours post-ingestion the concentration of DCA was low or negligible, so clinical toxicity is not likely to increase beyond this time (Figure 7). This contrasts to figure 4(d) where the elimination of propanil was prolonged and the concentration of DCA increased from admission until death. The reason for this difference is not apparent from this data, and because this kinetic profile appears atypical it was excluded from the regression analysis.

The median best-fit apparent elimination half life of propanil was 3.2 hours (95% confidence interval 2.6 to 4.1 hours); figure 6. The decay curves of the patients are noted to be approximately parallel, excluding two patients who died. The spread of these profiles most likely reflects differences in the bioavailable dose between patients. Two of the patients shown in figure 6 were known to receive exchange transfusion and their half-life did not appear to differ from the others.

A half-life was not calculated for DCA because the change in plasma concentration was highly variable. In some individuals the concentration increased during the sampling period while in the others it decreased. Therefore, a best-fit line would not be expected to represent the population. The increasing DCA concentrations at some late time points suggest that formation is more rapid than elimination, which may be more marked with higher concentrations of DCA, although this was not consistently observed.

**Figure 8:**
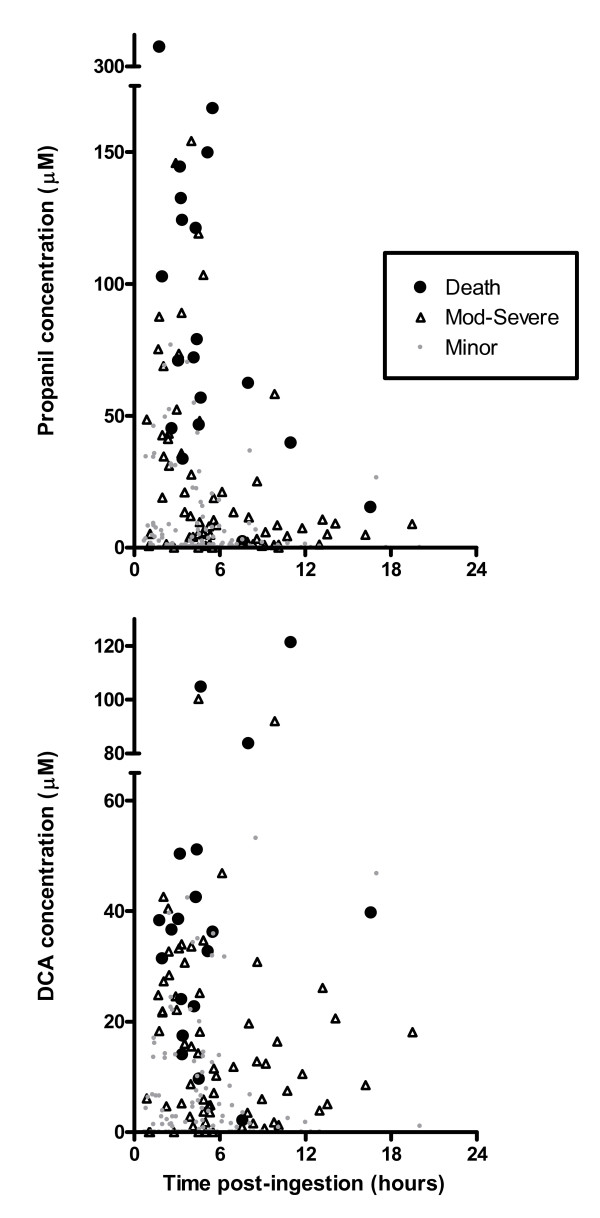
**Admission propanil and DCA plasma concentrations relative to the time post-ingestion and severity of poisoning**.

Admission plasma concentrations of propanil and DCA reflect clinical outcomes, as shown in figure 8, which supports the findings in the ROC curve (figure 3). With the exception of a few outliers, patients with the highest concentrations died and those with low levels demonstrated minor poisoning; both of these groups overlapped with patients manifesting moderate-severe poisoning. The relationship was less marked for DCA during the first 6 hours which probably relates to the time for bioconversion from propanil.

## Discussion

The mortality from acute intentional self-poisoning with propanil is 10.7% (95% CI 7.9–14.1%) making it the most lethal herbicide in Sri Lanka after paraquat. Clinical toxicity is characterised by cyanosis, acidosis and progressive end-organ dysfunction which are consistent with severe and prolonged methaemoglobinaemia. This reflects the elevated concentration of DCA that is subsequently metabolised to a hydroxylamine compound (figure 1) which appears to be the primary mediator of clinical toxicity. The most common specific treatments of methaemoglobinaemia were exchange transfusion and methylene blue and on the basis of clinical experience the former is considered more effective, but this could not be determined in this study. However, methylene blue was largely prescribed in low doses and administered as intermittent boluses which may be suboptimal with large propanil exposures. In the absence of controlled studies, the efficacy of these and other treatments is poorly defined. More research is required into the optimal management of acute propanil poisoning.

The previous kinetic data for propanil and DCA are limited to laboratory animal studies and a single patient. DCA and methaemoglobin are formed rapidly (within 2–3 hours [11,14]) in animals following injection of propanil, consistent with our findings in figure 4. By contrast, in a case report of human self-poisoning the peak DCA concentration was observed at 24 hours, which may relate to the co-ingestion with carbaryl.[4] The rate of bioconversion in our patients was variable, in particular the fatal case where it was prolonged, as shown in figure 4. Inter-individual differences in esterase activity due to genetics or co-exposure to other pesticides may explain such differences. However, there was no history of co-ingestion of other pesticides and BChE activity was normal (BChE activity was measured in patients where the elimination of propanil was prolonged; n = 18).

The specific CYP450 that *N*-hydroxylates DCA (figure 1) has not been characterised, but rat studies suggest that this reaction is saturable (K_m _= 120 μM [13] which might prolong the elimination of DCA in overdose. The highest concentration of DCA usually exceeded that of propanil; this probably relates to DCA being slightly less lipophilic than propanil (logP_o/w _= 2.69 and 3.07, respectively) and therefore likely to have a smaller Vd. It is also possible that in some of the patients the peak propanil concentration preceded admission and was therefore not observed.

The predominant clinical manifestation is methaemoglobinaemia. This occurs due to bioconversion of propanil to 3,4-dichlorophenylhydroxylamine, which is co-oxidised with oxyhaemoglobin (Fe^2+^) in erythrocytes to the ferric state (Fe^3+^). [11-14] Methaemoglobin is unable to bind and transport oxygen, inducing a relative hypoxia at the tissue level despite adequate arterial oxygenation. This leads to end-organ dysfunction, manifesting particularly as central nervous system depression, hypotension and acidosis.[23] However, the mechanism of toxicity may not be completely attributed to methaemoglobinaemia. In general, > 70% methaemoglobinaemia is associated with severe poisoning and death;[23] but in propanil exposures severe poisoning and death can occur when methaemoglobinaemia is as low as 40%.[3,5,6,9] It is therefore possible that toxic mechanisms other than methaemoglobinaemia may contribute to clinical outcomes.

Propanil may directly contribute to clinical toxicity. Rat studies have shown that if the esterases responsible for propanil metabolism (figure 1) are inhibited, propanil can induce poisoning in the absence of methaemoglobinaemia.[12] Haemolysis and anaemia have also been reported following acute propanil poisoning, [5-8] which has been attributed to the hydroxylamine metabolite.[15] Cellular toxicity induced by the hydroxylamine compound has been attributed to glutathione depletion,[24,25] but in another study intracellular glutathione was not altered.[26] Other demonstrable toxicity from the hydroxylamine compound includes lipoperoxidation,[27] myelotoxicity,[28,29] and immune dysfunction [30,31], however their clinical significance is not known.

Because the minimum toxic dose has not been determined and the potential for severe poisoning and death is high, all oral ingestions should be treated as significant and monitored for a minimum of 12 hours. Routine clinical observations are sufficient to detect the development of clinical toxicity. Clinical cyanosis and 'chocolate brown' coloured blood on white filter paper suggests methaemoglobinaemia and severity can be monitored with serial blood gases (including methaemoglobinaemia and lactate), if available. Pulse oximetry is not usually useful for monitoring methaemoglobinaemia and the response to treatment.[23] Most do not measure methaemoglobin and new pulse oximeters that can measure methaemoglobin have not yet been validated in a clinical setting with such potentially high readings.[32] It has been suggested that methaemoglobinaemia should be used to determine poisoning severity and that measuring the concentration of propanil is only useful for confirming exposure.[33] However, in our data propanil and DCA concentrations did reflect clinical outcomes (table 2, figure 3 and figure 8). Factors influencing the precision of this association include patient co-morbidities, documentation by the study doctors or differences in management such as the use of antidotes, although this cannot be determined from our data. This approximate concentration-response relationship reflects the proximity of propanil and its metabolites with the primary site of toxicity, the erythrocyte.

**Figure 9:**
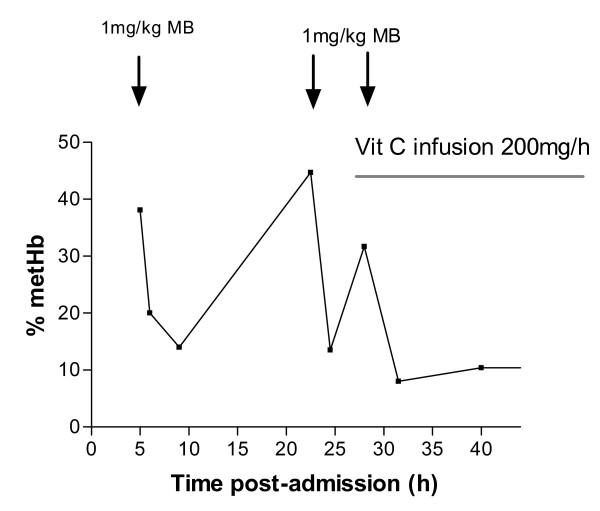
**Non-sustained reversal of methaemoglobinaemia by methylene blue (MB) in a patient with acute propanil poisoning despite multiple bolus doses**. The influence of ascorbic acid (Vit C) is not able to be determined from this data in the absence of kinetic data for DCA. (Adapted from Yamashita et al.[3]).

Until more data are available it seems reasonable to focus treatment on reversal of methaemoglobinaemia. Because the plasma concentration of DCA remains elevated methaemoglobinaemia will persist for a similar time. [3-5] Methylene blue has a half-life of 5 hours [34] which is shorter than that of DCA in many patients, figures 4 and 6. Rebound poisoning is therefore likely in severe cases from a bolus regimen (see figure 9 for example). This problem can be countered by administration of methylene blue as a constant infusion. This strategy has been reported as successful in dapsone poisoning [35-37] which similarly produces prolonged methaemoglobinaemia and has a much longer half-life than methylene blue.[38,39]

Methylene blue is intermittently unavailable and potentially of low efficacy in some patients so other treatments should be explored. Possibilities include acetylcysteine, toluidine blue and CYP450 enzyme inhibitors (eg. cimetidine). Acetylcysteine may act directly as an antioxidant or as a cysteine precursor that aids replacement of intracellular glutathione. *In vitro *studies have suggested that acetylcysteine directly reduces methaemoglobin, but very high concentrations were used.[40,41] These are equivalent to more than 500-times the dose used for paracetamol poisoning, which would be almost certainly associated with unacceptable adverse effects. We conducted an *in vitro *study using 4-DMAP (4-dimethylaminophenol [42])-induced methaemoglobin with a more reasonable concentration of acetylcysteine (1 mM) but did not find any significant reduction (data not shown). This has also been demonstrated by others *in vitro *[43] including a clinical study using sodium nitrite-induced methaemoglobin.[44] In contrast, *in vitro *studies with 3,4-dichlorophenylhydroxylamine have shown that cellular toxicity is reduced with 1 mM acetylcysteine or 2 mM ascorbic acid pre-treatment. [24]

Toluidine blue is another phenothiazine dye that in volunteer studies is more effective than methylene blue at reversing methaemoglobinaemia and has fewer adverse effects.[45] Co-administration of cimetidine and dapsone inhibits formation of the hydroxylamine metabolite of dapsone and methaemoglobin.[16,46-49] A similar effect is possible for propanil poisoning if DCA interacts with the same CYP450 enzymes, but no data are available and it is possible detoxifying enzymes might also be inhibited. There have been no clinical studies assessing whether such treatment has a role in clinical management.

Exchange transfusion is a popular treatment for methaemoglobinaemia in Sri Lanka, particularly if methylene blue is unavailable or ineffective. For some clinicians it is the preferred treatment for severe propanil poisoning.[10] The regimen is variable, but exchange of one unit every 1–2 hours for up to five cycles (usually three or less) was common in cases of severe poisoning. Exchange transfusion can improve oxygen delivery by donation of erythrocytes, although their function is temporarily impaired post-transfusion due to depletion of 2,3-DPG during storage. [50-53] At the same time free haemoglobin is removed.

Exchange transfusion may have additional benefits if it removes a sufficient amount of propanil from the body. The efficiency of this varies inversely with the proportion of poison located outside the vascular compartment, which depends on the Vd. The Vd of propanil is not known but is expected to be large given that both propanil and DCA are highly lipid soluble. The uptake and distribution of propanil in the channel catfish is extensive,[54] which also suggests that the Vd will be large in humans. From this it may be questioned whether the poison load will be markedly lowered by a few exchange transfusions. Further, transfusion reactions such as acute lung injury may occur which is of concern when tissue oxygenation is already impaired. Therefore, its use requires careful consideration.

In a single case of acute poisoning treated with combined haemodialysis and haemoperfusion there was a rapid decrease in propanil concentration during treatment (half-life = 1 hour). The authors of this case concluded that this treatment may be useful in patients with acute poisoning.[5] Unfortunately, the clearance was not measured directly. Because the half-life during enhanced elimination was not shorter than that in most of our patients (eg. figure 4) it does not appear to be particularly useful.

Given that poisoning manifests early post-ingestion and yet the time to death is usually greater than 24 hours (table 2 and figure 2) there are ample opportunities for interventions that prevent death. There are no controlled clinical or laboratory data available on the effect of any specific treatment in acute symptomatic propanil poisoning, so management is largely empirical. It seems reasonable that patients should be resuscitated and administered supportive care (particularly oxygen) as usual. Normoglycaemia should be ensured since adequate glucose concentrations are required for endogenous reduction and maximising the efficacy of methylene blue.[23]

Despite the lack of evidence, phenothiazine dyes such as methylene blue should be considered a first-line treatment for methaemoglobinaemia. It should be administered to symptomatic patients as frequent bolus doses of 1 mg/kg to a daily dose of up to 7 mg/kg (high doses may exacerbate methaemoglobinaemia and haemolysis),[45,55,56] although this maximum dose is conservative. This should be maintained by an infusion titrated to clinical response. If the response to this treatment is poor others should be considered, such as exchange transfusion. Other treatments including toluidine blue and acetylcysteine might also be considered as add on treatments if the patient continues to deteriorate clinically, but there are no data regarding efficacy. Monitoring of haemoglobin levels is recommended to detect haemolysis and folate supplementation may be necessary during the recovery phase if anaemia is significant.

Given the high mortality from acute propanil poisoning more research is needed to determine the optimal management. An area for urgent attention is to better describe the efficacy of methylene blue in terms of dose and regimen (intermittent bolus versus continuous infusion). Other studies for consideration include the relative efficacy of exchange transfusion, toluidine blue as well as add-on treatment with acetylcysteine and/or cimetidine. In addition to death, biomarkers should be measured as secondary endpoints for mechanistic data, including changes in methaemoglobinaemia, lactate concentration and markers of haemolysis. The effect of exchange transfusion on the kinetics of propanil and DCA should also be determined. Further, the influence of glucose-6-phosphate dehydrogenase deficiency (which is prevalent in parts of Sri Lanka [57,58]) on the response to methylene blue and clinical outcomes should be considered. It is generally recommended that methylene blue is not administered to patients with glucose-6-phosphate dehydrogenase deficiency because it is minimally effective, in addition to exacerbating methaemoglobinaemia and haemolysis.

## Conclusion

The case fatality of propanil is high compared to most other herbicides and current treatments appear to be inadequate. Clinical toxicity is primarily dose-related due to the metabolites. Because patients with limited clinical toxicity on admission may still die or develop severe poisoning, close monitoring is necessary. There is a substantial window of opportunity for interventions prior to death which should encourage further clinical research. Priorities include characterising the time course of methaemoglobinaemia, the efficacy of current and alternative treatments, and the means by which they may be optimised.

## Abbreviations

BChE: butyryl cholinesterase; CAS: Chemical Abstracts Service; CI: confidence interval; C_i_: initial concentration; C_t_: concentration after time t; CYP450: cytochrome P450 enzyme; DCA: 3,4-dichloroaniline; 4-DMAP: 4-dimethylaminophenol; 2,3-DPG: 2,3-diphosphoglycerate; GCS: Glasgow Coma Score; IQR: inter-quartile range; k: elimination rate constant; Km: Michaelis-Menten constant; P_o/w_: oil-water partition coefficient; LOQ: limit of quantification; LR: likelihood ratio; MABP: mean arterial blood pressure; MB: methylene blue; PK: pharmacokinetic; RCT: randomised controlled trial; ROC: receiver-operator characteristic; sens: sensitivity; spec: specificity; SpO_2_: Oxygen saturation by pulse oximetry; t_1/2_: half-life; TTP: time to present; TTD: time to discharge or death; Vd: volume of distribution; Vit C: vitamin C (ascorbic acid); XT: exchange transfusion.

## Competing interests

The authors declare that they have no competing interests.

## Authors' contributions

DMR extracted and analysed the data and drafted the initial manuscript. ME, NAB and AHD designed the clinical studies to which the patients were recruited. DMR, ME, FM and AHD contributed to the collection of data and coordination of these studies. RH and PE developed and conducted the laboratory analyses and take responsibility for these. All authors contributed to the final version of the manuscript and approve its submission. DMR coordinated the development of the manuscript, retains full access to the data presented, and had final responsibility for the decision to submit for publication.

## Pre-publication history

The pre-publication history for this paper can be accessed here:


